# The multiple roles of fatty acid handling proteins in brain

**DOI:** 10.3389/fphys.2012.00385

**Published:** 2012-09-28

**Authors:** Valentine S. F. Moullé, Céline Cansell, Serge Luquet, Céline Cruciani-Guglielmacci

**Affiliations:** Université Paris Diderot, Sorbonne Paris Cité, BFA, EAC 4413 CNRSParis, France

**Keywords:** fatty acids, brain, FATP, FABP, CD36, GPR40, neuronal differentiation, cognition

## Abstract

Lipids are essential components of a living organism as energy source but also as constituent of the membrane lipid bilayer. In addition fatty acid (FA) derivatives interact with many signaling pathways. FAs have amphipathic properties and therefore require being associated to protein for both transport and intracellular trafficking. Here we will focus on several FA handling proteins, among which the fatty acid translocase/CD36 (FAT/CD36), members of fatty acid transport proteins (FATPs), and lipid chaperones fatty acid-binding proteins (FABPs). A decade of extensive studies has helped decipher the mechanism of action of these proteins in peripheral tissue with high lipid metabolism. However, considerably less information is available regarding their role in the brain, despite the high lipid content of this tissue. This review will primarily focus on the recent studies that have highlighted the crucial role of lipid handling proteins in brain FA transport, neuronal differentiation and development, cognitive processes and brain diseases. Finally a special focus will be made on the recent studies that have revealed the role of FAT/CD36 in brain lipid sensing and nervous control of energy balance.

## Introduction

All mammalian cells require energy and nutrient supplies for their proper functioning. Aside of glucose and protein, which represent respectively the major source of energy for both the brain and the red cells, and the critical source of nitrogen, lipids are the most energetic source. Lipids are used almost exclusively by the body during long term energy deprivation but represent also the major component of plasma membranes. Therefore a sustained supply of lipids is essential for the proper development, differentiation, and metabolism of most cells. Fatty acids (FAs) are amphipathic molecules that require specific buffering processes to circulate in the plasma and cross the plasma membrane. Indeed, protein-based transmembrane translocation and intracellular FA binding proteins allow lipid-based molecules to access all sub cellular compartments. Although the fine mechanism is still elusive, this is a fundamental process by which a lipid-based molecule could enter the cell and modulate signaling pathways and the activity of transcription factors. Some FA handling proteins such as the fatty acid translocase FAT/CD36, fatty acid transport proteins (FATPs), and lipid chaperones fatty acid-binding proteins (FABPs) have been extensively studied in peripheral tissues but considerably less information is available regarding their role in the brain. This review attempts to cover their role in brain FA transport, neuronal differentiation and development, cognition, and energy homeostasis.

## FA handling proteins in blood-brain transport

Brain is surrounded with the blood-brain barrier (BBB) which provides protection against the entry of harmful substances and allows the removal of waste produced. The BBB is composed of four cell types including endothelial cells, pericytes, astrocytes, and neurons altogether composing the neurovascular unit (Hawkins and Davis, 2005). An anatomical particularity of the BBB is that brain microvessel endothelial cells are connected by strong tight junctions that direct plasma substances into transcellular routes and reduce the paracellular diffusion of solutes and macromolecules (Benarroch, 2011). FA transport across the BBB relies on two distinct mechanisms: a passive diffusion through the lipid bilayer called FA flip-flop, mostly due to FA lipophilic properties (Hamilton and Brunaldi, 2007), and the facilitated transport of FAs that requires the presence of saturable FA transporters. FATPs, FAT, and FABPs have been identified in endothelial cells of the BBB. A recent study, using human brain microvessel endothelial cell (HBMEC) monolayers, demonstrated that among the different FA transporters expressed, FATP-1 (SLC27A1), and FATP-4 (SLC27A4) are the most abundant in HBMEC while FAT/CD36 insures the majority of FA transport (Mitchell et al., 2011). FATPs are a family of six membrane proteins; they have one transmembrane domain with an extracellular FA binding site and an intracellular ATP binding domain with acyl-CoA synthetase activity (Mitchell and Hatch, 2011). FAT/CD36 is a membrane protein discovered in 1993 in rat isolated adipocytes (Abumrad et al., 1993) that is expressed in many tissues and binds many ligands: thrombospondin, an endogenous inhibitor of angiogenesis (Dawson et al., 1997), saturated and unsaturated FAs with an affinity in the nanomolar range (Baillie et al., 1996), oxidized low density lipoproteins (Puente Navazo et al., 1996) and pathogenic agents such as *Plasmodium falciparum* (Baruch et al., 1999).

Besides their presence in the BBB, lipid handling proteins are also largely expressed in different brain areas (Schaffer and Lodish, 1994; Utsunomiya et al., 1997; Owada, 2008) and are involved in both brain development and adult neurogenesis.

## FA handling proteins in brain development and neuronal differentiation

FAs, notably polyunsaturated FAs (PUFAs), are necessary for normal development of the central nervous system (CNS) (Neuringer et al., 1988). Indeed, brain has a lipid content of about 50% of its dry weight suggesting an essential role of lipids in membrane constitution and signaling pathways (Edmond, 2001; Rapoport et al., 2001). Many psychiatric diseases such as schizophrenia, depression, Parkinson's disease or Alzheimer dementia (AD) are associated with PUFA deficiency (Salvati et al., 2006; Watanabe et al., 2007; Iwayama et al., 2010). Because PUFAs are lipophilic molecules, their intracellular trafficking needs chaperones proteins called FABPs. Among the 10 members of FABP family, three of them have been shown to participate in the development and function of the adult brain (Liu et al., 2010): Heart-FABP (H-FABP; FABP-3), Epidermal-FABP (E-FABP; FABP-5), and Brain-FABP (B-FABP; FABP-7). H-FABP is expressed during the late phase of brain development. It could participate to neurite formation and synapse maturation, and supports neuronal function in adult age (Sellner et al., 1995; Owada et al., 1996). E-FABP is more expressed in neurons before and after birth suggesting a role in neuronal differentiation. Indeed, its differential temporal expression during development is consistent with its proposed role in the transport of long chain FAs and/or other lipophilic ligands during neuronal differentiation and axon growth (Liu et al., 2008). B-FABP is specifically expressed in radial glial cells implicated in neuron migration during brain development (Feng et al., 1994) and its expression is partially regulated by the presence of migrating neurons (Feng and Heintz, 1995; Anthony et al., 2005). Knockout studies show that H-FABP knockout mice present a reduction in both incorporation of arachidonic acid (AA; 20:4 n-6) and proportion of total ω-6 FAs in the major phospholipid classes, suggesting a role for H-FABP in ω-6 FAs uptake and metabolism in the developing brain (Murphy et al., 2005). B-FABP knockout leads to a decreased number in astrocytes, neural stem cells and early progenitor cells in developing brain suggesting that B-FABP plays an important role in neurogenesis by maintaining the pool of neural stem/progenitor cells notably in hippocampus (Watanabe et al., 2007). Contrary to other FABPs, E-FABP knockout mice have no brain phenotype suggesting a compensatory phenomenon with other brain FABPs (Owada et al., 2002). Besides their important roles in brain development, FABPs may also contribute to the proliferation of neural progenitor cells (Boneva et al., 2011). FABPs are expressed in astrocytes where they serve as chaperone proteins to drive PUFAs to the nucleus and participate to the regulation of gene expression, membrane synthesis and β-oxidation and to the controlled release of PUFAs acting as paracrine signals for newborn neurons (Yamashima, 2012).

In addition, long-chain acyl-coA synthase 4 (LACS4; ACSL4) has been shown to regulate neuronal differentiation. LACS4 catalyzes the formation of acyl-CoA from FAs, ATP and CoA and plays an important role in arachidonate metabolism (Kang et al., 1997). Docosahexaenoic acid (DHA; 22:6 n-3) and AA are important supplements for neuronal differentiation (Kan et al., 2007), in addition cell neogenesis requires a significant amount of FAs to produce the lipid bilayer of the cytoplasmic membrane. It is possible that LACS4 deficiency might interfere with AA re-esterification into the neuronal membrane during differentiation. Moreover, LACS4 knockout in mouse embryonic stem cells attenuates neuronal differentiation and neurite outgrowth induced by growth factors (Cho, 2012).

Finally, brain expression of GPR40 (FFAR1) suggests a role in detection of PUFAs and mediation of their metabolic or signaling effects. GPR40 was discovered in 1997 by Sawzdargo et al. (1997), and belongs to the G-protein coupled receptors (GPCR) family which gathers many proteins with seven transmembrane domains. The binding of PUFAs to GPR40 initiates an intracellular cascade involving phospholipase C and phosphatidylinositol (Briscoe et al., 2003; Itoh et al., 2003; Kotarsky et al., 2003). In mice, GPR40 is expressed in the olfactory bulb, striatum, hippocampus, midbrain, hypothalamus, medulla oblongata, cerebellum, and cerebral cortex (Nakamoto et al., 2012). In humans and monkeys, GPR40 is expressed throughout the brain in neurons, and its presence in astrocytes of the subventricular zone and subgranular zone indicates a potential involvement in the regulation of adult neurogenesis (Ma et al., 2007). Ma et al. showed that GPR40 was upregulated in astrocytes and newborn neurons after transient global cerebral ischemia in the neurogenic niche of adult monkey hippocampus (Ma et al., 2008). This phenomenon is associated with the increase in DHA content which might stimulate the generation and differentiation of newborn neurons through a GPR40 upregulation and CREB (c-AMP response element-binding) activation (Yamashima, 2012).

Consequently, abnormalities in FA handling protein expression can affect the normal growth of the CNS which in turn can lead to cognitive impairment (Bourre, 2004; Abumrad et al., 2005) and psychiatric diseases such as schizophrenia (Watanabe et al., 2007).

## FA handling proteins and cognitive processes

Among the different mechanisms, FA handling proteins could modulate cognition by influencing brain FA transport and therefore by modulating supply of the needed FAs to the brain. For example AA and DHA are the major constituents of neural cell membrane phospholipids which are derived respectively from the dietary precursors: linoleic acid and α-linolenic acid. *De novo* synthesis is very limited in Mammals, so brain composition and function can be sensitive to dietary influences and brain transport. B-FABP deletion induces a decrease by 4% in the content of DHA and an increase by 4% in the content of AA in total neonatal brains (Owada et al., 2006). B-FABP null mice have no impairment of spatial learning/memory but the plus-maze test shows a behavioral alteration that suggests an enhancement of anxiety. This study demonstrated that the change in DHA and AA content at the neonatal stage, although small, could be critical in the establishment of emotional disorders in adult (Owada et al., 2006). Recently, it has been shown that CD36 null mice have decreased levels of monounsaturated FAs in several brain phospholipid pools (Song et al., 2010). CD36 null mice show normal levels of anxiety and exploration patterns of novel environment. However Abumrad et al. noticed a behavioral difference in the eight radial arm mazes between CD36 null and WT mice, reflecting a deficit in learning ability (Abumrad et al., 2005). It is not clear if these changes are related to altered brain phospholipid composition.

In addition, some lipid handling proteins are able to modulate neurotransmission. In a recent study, Nakamoto et al. showed that DHA-induced antinociception via β-endorphin release may be mediated through GPR40 signaling in the supraspinal area (Nakamoto et al., 2012). They suggested that GPR40 signaling activated by DHA or GW9508 (GPR40-selective agonist) may lead to a Ca^2+^ influx and an acceleration of β-endorphin release in nerve ending particles or synaptosomes. In fact, intracerebroventricular injection of DHA and GW9508 significantly reduced formalin-induced pain behavior, and those effects were inhibited by pretreatment with anti-β-endorphin antiserum (Nakamoto et al., 2012). Moreover, H-FABP and the intracellular form of dopamine D2 receptor (D2R) co-immunoprecipitate in brain extracts and colocalize immunohistochemically in the dorsal striatum (Takeuchi and Fukunaga, 2003; Shioda et al., 2010). Dopaminergic system and particularly D2R has been implicated in reward mechanisms and locomotor control in the brain. H-FABP null mice have D2R dysfunction showed by lower responsiveness to metamphetamine-induced locomotor sensitization and enhanced haloperidol-induced catalepsy compared with WT mice. H-FABP is expressed in acetylcholinergic interneurons and of glutamatergic neuronterminals in the dorsal striatum of mouse brain but is absent in dopamine neuron terminals and spines in the same region. H-FABP null mice exhibit no change in total D2R protein levels compared with WT, suggesting that H-FABP does not alter D2R stability or turnover in the striatum. D2R dysfunction is associated with an increase in KCl-induced acetylcholine and glutamate release, suggesting that H-FABP regulates dopamine-regulated Ach and Glu release through D2R in the striatum (Shioda et al., 2010).

Finally, genomic or RNA analysis have shown that some FABP genes are associated with psychiatric disorder, e.g., LACS2 (ACSL2) expression is upregulated in cortical regions of rat model of depression (Setnik and Nobrega, 2004). Moreover, expressional changes and genetic variants of B-FABP are observed in mouse models and in human schizophrenia (Iwayama et al., 2010).

## FA handling proteins as potential therapeutic targets in brain disease

As described above, FA handling proteins have an important role in many brain functions. Increasing evidences indicate that a dysfunction or a deregulation in brain FA handling protein expression or action could play a pathogenic role in many brain diseases. In addition CD36 and LACS can be considered as potential therapeutic targets to treat AD, bipolar disorders or Refsum disease (RfD), which is an autosomal recessive neurologic disorder of the lipid metabolism.

In atherosclerosis and AD, deposition of oxidized low-density lipoproteins and Amyloid-β peptide (Aβ) triggers an inflammatory response through a heterodimer of Toll-like receptors 4 and 6 regulated by signals from the scavenger receptor CD36 (Stewart et al., 2010). Aβ has profound deleterious vascular effects mediated for the most part by the reactive oxygen species (ROS) produced by the NADPH oxidase enzyme. Recently it has been reported that these Aβ effects are not observed in CD36 null mice (Park et al., 2011). Park et al. suggest that CD36, localized mainly to endothelial cells, may interact with Aβ to activate NADPH oxidase, triggering vascular oxidative stress and cerebrovascular dysfunction. In addition, CD36-mediated assembly of hp130Cas complexes is an essential part of the Aβ signaling that induces microglia migration to sites of Aβ deposition. Recently, the CD36 ligand ursolic acid was identified as a potential therapeutic agent for AD via its ability to block Aβ-CD36 interactions (Wilkinson et al., 2011).

In bipolar disorders, valproic acid is used as a treatment by acting as a non-competitive inhibitor of brain microsomal LACS (Bazinet et al., 2006). Bazinet et al. showed that this treatment induced a decrease of arachidonoyl-CoA production by reducing the brain AA cascade. Moreover, the murine LACS (mLACS; ACSL2) have been involved in RfD (Kee et al., 2003). RfD is biochemically characterized by the excessive accumulation of phytanic acid in tissues and body fluids due to the deficiency of phytanoyl-CoA α-hydroxylase (PAHX) which normally interacts with multiple proteins. Kee et al. propose that, in the RfD, lack of PAHX interaction with mLACS in neural tissue may affect the role of mLACS on lipid metabolism leading to the development of neurological symptoms.

In summary, FA handling proteins are implicated in many brain functions and potential diseases. However little information were gathered regarding the implication of these proteins in the nervous control of energy balance.

## Implication of FA handling proteins in brain lipid sensing and nervous control of energy balance

Since free FAs are not primary metabolic fuel for neurons, their role in brain metabolism remained questionable for decades. However, accumulating evidence demonstrated that FAs are used in specific areas of CNS as cellular messengers which inform “FA sensitive neurons” about energy status of the body (Migrenne et al., 2011). This phenomenon, named “brain lipid sensing” as an analogy with glucosensing, is involved in the control of feeding behavior, hepatic glucose production, and insulin secretion (Obici et al., 2003; Cruciani-Guglielmacci et al., 2004). Increasing evidence show that CD36 may act as receptor, rather than a transporter, for long chain FAs. In fact, El-Yassimi et al. showed that linoleic acid binding on CD36-positive gustatory cells induced src-kinases phosphorylation, calcium intracellular concentration changes and serotonin and dopamine release (El-Yassimi et al., 2008; Gaillard et al., 2008). These events could send nervous signals to the brain and induce peripheral modifications. In the brain, Levin et al. showed that different neuronal groups in hypothalamus responded differently to glucose or oleic acid (Le Foll et al., 2009). Indeed some neurons are able to increase or decrease their activity in response to oleic acid. The addition of sulfosuccinimidyl oleate (CD36 inhibitor) or triacsin C (ACS inhibitor) on freshly differentiated neurons lead to alteration of the excitatory or inhibitory effects, indicating the involvement of these proteins in central FA detection (Le Foll et al., 2009). In addition, studies suggest that hypothalamic FA sensitive neurons are dependent on the metabolic state and display different responses during fasting versus overfed state (Le Foll et al., 2009; Migrenne et al., 2011). Energy status could also modify the FA transport to the CNS regarding to brain needs. For example, fasting induces a 30% decrease in glucose uptake and a 300% increase in palmitate uptake in hypothalamus, which is correlated with plasma changes (hypoglycemia and plasma FA increase) (Kasser et al., 1986). In our laboratory, we showed that hypothalamic CD36 expression was decreased about −40% after 48 h-fasting and increased about +36% after 2 months of high fat diet, suggesting that the metabolic status can modulate brain CD36 expression (unpublished observations). Variations of CD36 expression could modulate lipid signaling in the brain and participate to the regulation of energy homeostasis. Dysfunction of these FA sensitive neurons could provide, at least in part, an early mechanism underlying the impairment of neural control of energy and glucose homeostasis and the development of obesity and type 2 diabetes in predisposed subjects (Elmquist and Marcus, 2003).

Moreover, recent studies show that hypertriglyceridemia, observed in obesity, is associated with cognitive impairments (Farr et al., 2008). Obese mice show impaired acquisition in cognitive paradigms like elevated plus-maze test, a phenomenon that can be corrected by lowering triglycerides. In addition, rats on high energy diet show a reduced hippocampal synaptic plasticity and an impairment of cognitive function (Stranahan et al., 2008). We can suppose that a defect in brain lipid sensing ability relayed by specific FA handling protein deficiency could induce cognitive impairment. For example, CD36 null mice show a deficit in learning ability (Abumrad et al., 2005) whereas there were no significant differences in brain AA and DHA compared to control mice suggesting that other mechanisms than brain composition are involved in this phenotype.

## Conclusion

A better understanding of the central role of FA handling proteins could provide important clues for the identification of new therapeutic targets for the prevention and treatment of both metabolic and cognitive diseases.

### Conflict of interest statement

The authors declare that the research was conducted in the absence of any commercial or financial relationships that could be construed as a potential conflict of interest.
